# The potential of including the microbiome as biomarker in population-based health studies: methods and benefits

**DOI:** 10.3389/fpubh.2024.1467121

**Published:** 2024-10-23

**Authors:** Florence E. Buytaers, Nicolas Berger, Johan Van der Heyden, Nancy H. C. Roosens, Sigrid C. J. De Keersmaecker

**Affiliations:** ^1^Transversal Activities in Applied Genomics, Sciensano, Brussels, Belgium; ^2^Health Information, Sciensano, Brussels, Belgium

**Keywords:** microbiome, population-based health studies, population, cohort, metagenomics, biomarkers

## Abstract

The key role of our microbiome in influencing our health status, and its relationship with our environment and lifestyle or health behaviors, have been shown in the last decades. Therefore, the human microbiome has the potential to act as a biomarker or indicator of health or exposure to health risks in the general population, if information on the microbiome can be collected in population-based health surveys or cohorts. It could then be associated with epidemiological participant data such as demographic, clinical or exposure profiles. However, to our knowledge, microbiome sampling has not yet been included as biological evidence of health or exposure to health risks in large population-based studies representative of the general population. In this mini-review, we first highlight some practical considerations for microbiome sampling and analysis that need to be considered in the context of a population study. We then present some examples of topics where the microbiome could be included as biological evidence in population-based health studies for the benefit of public health, and how this could be developed in the future. In doing so, we aim to highlight the benefits of having microbiome data available at the level of the general population, combined with epidemiological data from health surveys, and hence how microbiological data could be used in the future to assess human health. We also stress the challenges that remain to be overcome to allow the use of this microbiome data in order to improve proactive public health policies.

## 1 Introduction

**Figure 1:**
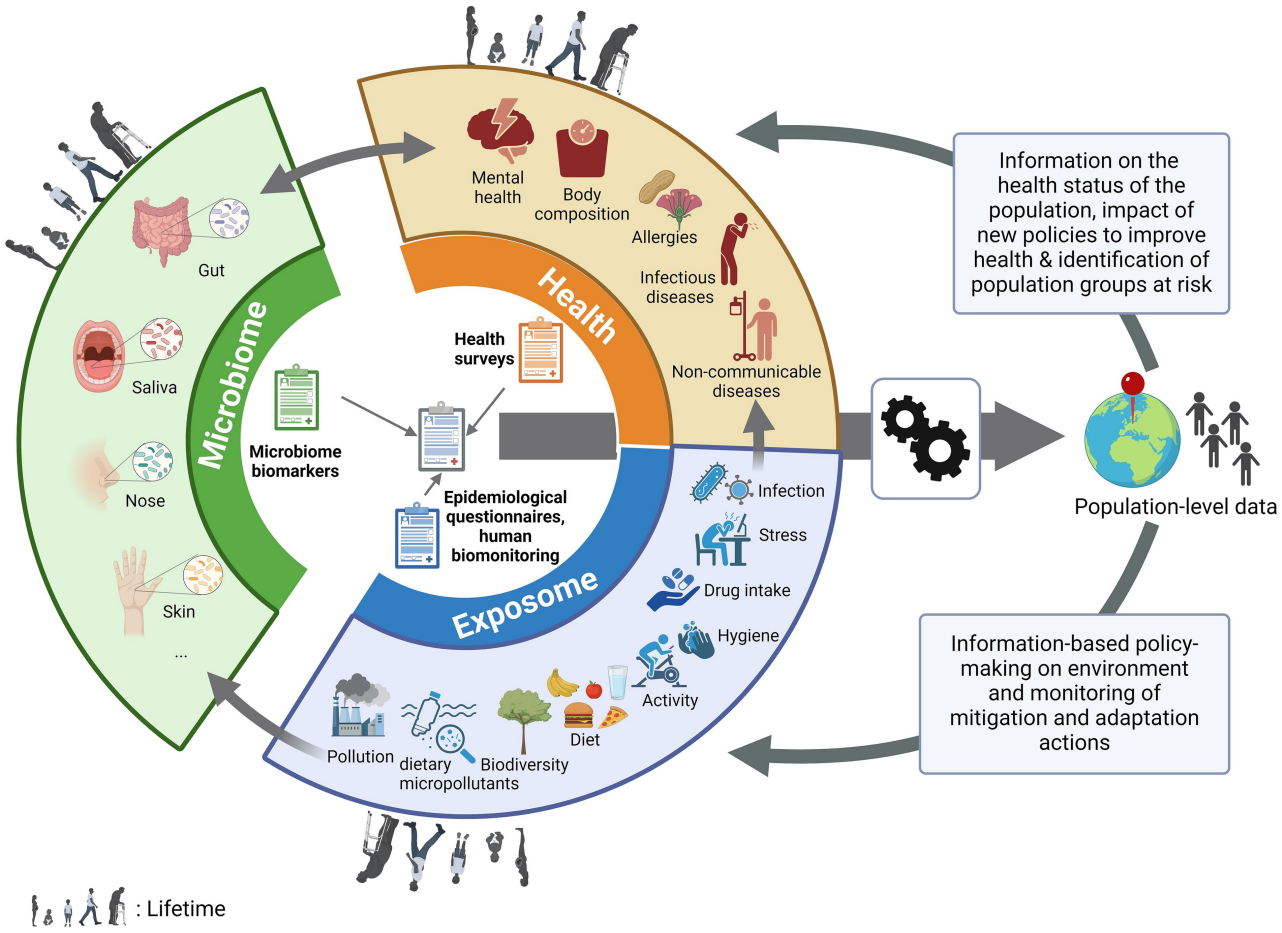
The pivotal place of the microbiome in population-based health studies. Biological data from non-invasive microbiome (gut, oral, nasal, skin…) testing performed at population-scale, can be associated with information from health, epidemiological surveys and human biomonitoring, with information on environmental and health behavior parameters (exposome, e.g., diet, pollution, stress….) affecting the population through life as well as the prevalence or development of health conditions (e.g., body composition, allergies, diseases, mental health…). After (bio)informatic and statistic processing, this population-level data could be used to drive proactive public health policy by giving information on the health status of the population (and therefore monitor the impact of new policies to improve health) or to identify population groups at risk for specific diseases or health problems and by allowing information-based policy-making on the environment and monitoring of the mitigation and adaptation actions put in place.

In recent years, several large studies have been carried out sequencing thousands of human genomes (1, 2). However, it is now known that humans are holobionts, or meta-organisms, living in the presence of thousands of microbes on different parts of the body, such as the gut, vagina, skin, mouth and nose (3, 4), constituting a second genome (5). These organisms have a symbiotic interaction with our bodies and play an important role in our health from birth, for example by programming our immune system, providing nutrients for cells and preventing colonization by pathogens (6). Over the past decade, alterations in the microbiome have been associated with the occurrence of a plethora of diseases and conditions, including diabetes, obesity, inflammatory bowel disease, autism and mental health (7, 8). Microbiota transplantation is now even being used as a validated treatment for recurrent *Clostridium difficile* infections (9). However, the causal effects of the microbiome on health outcomes, or vice versa, are still not fully documented or understood (10–12). Studies have shown that the microbiome, which is even thought to be initiated in the prenatal state (13), is also shaped by exposures throughout our lives (i.e., our exposome). Our microbial diversity begins to expand after the introduction of solid foods and then continues to vary according to health behavior (14, 15), including the diet, stress or activity levels (16), antibiotic use (17), but also our environment (18, 19), including exposure to pollution and environmental contaminants (20, 21) and the biodiversity in the surrounding landscape (22). Infection by a pathogen (23) or non-communicable diseases have also been associated with changes in the microbiome, e.g., metabolic syndrome (24), cancer (25) or pulmonary damage after pollution exposure (26). In addition, the microbiome could predict the severity of the infection, as reported for COVID-19 (27–29). The microbiome has also been shown to indicate how individuals respond to certain drugs, e.g., how cancer patients respond to chemotherapy (30). These recent data suggest that microbiome analysis, when combined with epidemiological data, has the potential to provide valuable information for assessing population health. Furthermore, as the microbiome is influenced by the exposome, the microbiome could also be used to assess environmental exposures. Therefore, combining the determination of the microbiome with population-based epidemiological data (e.g., from national health examination surveys, food consumption surveys or national cohorts) would make it possible to obtain information on the general health status of the population as well as the impact of environmental and health behavior parameters (exposome) on this health or to identify population groups at risk for specific diseases or health problems through biological sampling (Figure 1) (31, 32). Hence, through further research combining epidemiological and microbiome evaluations, microbiome data has the potential to be used as biomarker, i.e., a kind of proxy or measurable indicator of health and exposure determinants in the general population. This would provide relevant authorities with important insights to drive a proactive public health policy (Figure 1).

In this paper, we discuss various practical considerations and benefits of including the microbiome in population-based health studies. We present the potential samples that can be collected to analyze the human microbiomes and how this sampling can be standardized for large-scale public health studies. We describe the different technologies now available to analyze the microbiome of these samples and how this analysis will affect the information that can be obtained from the study. We then discuss how this approach can be implemented in population-based surveys and cohorts, using examples of studies that could benefit from the addition of the study of the microorganisms that live in and on us. Finally, we highlight the opportunities and challenges of including the microbiome in such population-based health studies.

## 2 Considerations on microbiome sample type selection and collection

The purpose of the research will determine the type of biological sample. The skin or vaginal microbiome may not answer the same biological questions as the microbiome from the nose, mouth or gut. Therefore, the specific microbiome community to be studied must be carefully chosen. Gut and oral microbiomes are very diverse and have been most studied in relation to diseases and environmental exposures. They are therefore promising options for use in population-based health studies. The collection of feces (i.e., stool) to study the gut microbiome is non-invasive, allows self-sampling and has therefore been documented in large-scale studies (33, 34). The oral cavity has been described as the second largest human environment after the gut in terms of diversity and abundance of microorganisms (35). Samples of the oral microbiome are also easy to collect and preserve, although the exact sample type or location (saliva, tongue swab, biofilm at tooth surface, subgingival plaque…) may give different results (36). Saliva is the easiest to collect by the participants without external intervention and offers the possibility to also investigate the human genome or epigenome (37). Finally, nasal or skin swabs have been shown to be relevant when investigating environmental parameters such as air pollution or greenness and biodiversity levels of the surrounding landscape (38). The lung microbiome can also be of interest when studying respiratory diseases and exposure to air pollution (39–41), although the sampling collection methods are commonly invasive. The collection of sputum is the least invasive sampling of the lung microbiome, although the microbiome obtained is inheritably contaminated by the oral microbiome, and therefore does not represent the ‘pure’ respiratory microbiome. Furthermore, healthy participants may not have sufficient phlegm, which can hinder sampling (42, 43). Once the sample type has been selected, the sampling method (e.g., self-sampling or interviewer-led) needs to be determined. To conduct large-scale studies, self-sampling, preferably without the need for the presence of a health practitioner, as well as the existence of a standardized sampling protocol, will be crucial to limit bias, as has been extensively reviewed (44, 45). This standardization includes the collection [e.g., for nasal swabs, the depth (46)], the timing [e.g., first feces of the day (47)] as the microbiome is affected by circadian changes (48), the collection tubes and the transport and storage conditions (44, 45). Importantly, in Europe, in the context of the General Data Protection Regulation (GDPR), each donor must be (pseudo-)anonymized and the samples must be carefully stored in a biobank (49, 50) and linked to the donor consent. Moreover, although studies have reported relationships between the microbiome and human genome markers, making a combined collection interesting (51), this may raise some ethical issues. Notwithstanding, for GDPR reasons, it is common practice to remove all human genetic data from publicly stored microbiome data to avoid re-identification of the donor.

## 3 Approaches to analyze the microbiome

Until recently, microorganisms have mainly been studied in isolation after being cultured on plates. This is a tedious task for complex environments and communities, and not all organisms living in the human microbiomes can be cultured (52), including viruses. In recent decades, new culture-independent methods have been developed that allow a better understanding of the diversity of the human microbiomes in a single test. These methods are based on sequencing the nucleic acids (mostly DNA) extracted from the microbiome sample. The quality, abundance and unbiased representation of the microbes in the genetic material extract is important to consider for standardization and data comparison (53).

The most widely used method of microbiome analysis is metabarcoding or sequencing of the 16S rRNA subunit gene, a highly conserved gene shared by bacteria and archaea, but presenting hypervariable regions that allow one bacterium to be distinguished from another. Two methods can be used, based on a selection of hypervariable regions of the gene (V1 to V9, of which V3-V4 are the most frequently selected), which may differ in resolution, or based on the sequencing of the entire gene. However, when only regions of the gene are sequenced, it has been reported that this differentiation is limited to accurately determine the genus level for most species (54, 55), whereas the species or strain level may be required to determine accurate biomarkers from the microbiome (56). The sequencing of the entire gene may improve species level identification but still only allows taxonomic classification and not functional analysis (55). Importantly, the use of different hypervariable regions leads to different results (44, 57), which may also bias conclusions from meta-analyses (58). Moreover, this method does not report the presence of fungi (which could be analyzed by the ITS region), eukaryotes (which could be analyzed using the 18S rRNA subunit), but also viruses, which are not targeted by any metabarcoding method (59).

An alternative method that has been developed is shotgun metagenomics or the sequencing of all nucleic acids in the sample, which theoretically allows the entire genome of all the species in the sample to be obtained, including non-bacterial species (60). This includes the information on the different taxa present at the strain level (61), but also allows the functional analysis through the genes present in each genome. In particular, the presence of antimicrobial resistance genes can also be analyzed which is of particular importance for public health. Recently, a team of scientists even defined statistically relevant associations of the donor body mass index at SNP (single-nucleotide polymorphism) level, which is only possible when the entire genome of each microorganism in the sample is sequenced (56). As this method is still expensive, it could be used in a public health setting for pioneering studies to define markers to look for, but may not be feasible for a whole cohort or population, depending on the budget available. This method also allows access to the sequence of the genetic material from the donor as well, which could be of interest for some studies targeting the human DNA, making it more cost-effective. If biomarkers are defined at species, gene or SNP level after sequencing, more targeted methods can be used to test for them (62), providing a much faster and less expensive alternative. However, target-based methods could not be used when the microbiome change of interest is a variation in diversity, richness or evenness, requiring a view of the entire microbiome at once.

Finally, promising alternative methods are being developed. For example, optical mapping, which is based on the examination of fluorocoding labels on the DNA with a high-resolution microscope to visualize structural variations within a microbial genome, allows species identification at a lower cost and in a shorter time frame (63). However, it does not provide information on the gene content. Specific genetic markers can be detected at a lower cost without the need for shotgun sequencing using hybridization capture sequencing (64), but the gene cannot be linked to the genome and species harboring it. Depending on the scientific question to be answered, multiple microbiome characterization methods could be coupled together to provide a complete view of the samples and limit the bias of each individual method (65). Alternatively, RNA sequencing using metatranscriptomics may also be of interest to study RNA viruses, which have also been reported to be present in microbiomes (66). It is important to note that the handling of the sample, storage, DNA extraction, library preparation, sequencing and data analysis all introduce bias. Therefore, regardless of the method chosen, in addition to including appropriate controls during the analysis (45), the data must be correctly stored in a database with accurate annotation and metadata to allow further analysis and comparison between different studies or approaches (67, 68). Furthermore, as reviewed elsewhere (45), microbiome analysis requires appropriate bioinformatics and biostatistics.

## 4 Including the microbiome as a potential biomarker in population-based health studies –examples of possible applications

As discussed in the introduction, non-population-based studies have revealed various associations between the microbiome and various aspects of health (as reflected in Table 1), and between environmental exposures and microbial composition, diversity, and function (Table 1), although the biomarkers that have been described are not yet validated at the whole population level. Conducting microbiome studies at the population level offers additional advantages, including broader representation (general population), longitudinal analysis (temporal patterns, causal relationships, i.e., cohorts), large-scale data (higher reliability), and translational impact (actionable insights). Once biomarkers can be clearly defined, by integrating the biological measures from the microbiome data that can be correlated with health disturbance, with the data typically collected during population-based health studies (questionnaires, chemical exposure), we could assess the population’s health status and the impact of environmental and health behavior parameters on that health (Figure 1). This has the potential to improve our understanding of disease etiology, risk factors, and prevention strategies, ultimately contributing to improved population health outcomes through information-based policy making (Figure 1; Table 1).

**Table 1:** Examples of scientific publications focused on various exposures, including microbiome samples of smaller populations and their main observations, as biological evidence obtained from the microbiome, that could be implemented in future population-based health studies in support of policy when biomarkers are clearly defined.

Exposure	Examples (references)	Microbiome investigated (sample type)	Sample size (number of subjects included)	Analysis method	Observations	Possible contribution to policy in the future
Food consumption	David et al., 2014 (69)	Gut (feces)	11	16S rRNA V4	Short-term alterations of the microbiome after short-term shift to animal-based diet or plant-based diet	Monitor mitigation actions to promote healthy diet (such as nutri-score)
	Oliver et al., 2021 (70)	Gut (feces)	26	Shotgun metagenomics	Increasing the intake of dietary fiber over the course of two weeks significantly altered the composition of individual gut microbiomes	
Chemical exposure	Abdelsalam et al., 2020 (71)	Review based on gut microbiome studies	Not applicable (review)	Favors shotgun metagenomics to 16S rRNA	The human microbiome has a metabolizing potential on xenobiotics but the xenobiotics can also alter the composition of the microbiome, leading to dysbiosis	Recommendation from the European Food Safety Authority to use the human gut microbiota for risk assessment analysis of cumulative xenobiotic exposure (62)
	Thompson et al., 2022 (20)	Gut (feces)	124	Shotgun metagenomics	Association between lifetime exposure to organochlorine compounds (including pesticides), PFAS and mercury and alterations in the gut microbiome	Mitigation actions to limit exposure to contaminants, monitoring of effects of those mitigation actions on the population in combination with other well-validated clinical measures
	Fournier et al., 2023 (72)	Gut (infant *in vitro* gut model)	4	16S rRNA V3-V4	Exposure to polyethylene microplastics of an infant *in vitro* gut model induced a microbial shift and increase of abundance of potential harmful pathobionts	
Anxiety	Radjabzadeh et al., 2022 (73)	Gut (feces)	> 2,500	16S rRNA V4	Association between the gut microbiome (13 microbial taxa) and depressive symptoms	Biological measure to associate with mental health questionnaires at population level
Environment/biodiversity/air pollution	Hanski et al., 2012 (22)	Skin (forearm)	118	16S rRNA V1-V3	Correlation between the environmental biodiversity, skin microbiota and allergies	Biological measure associated with the health status and the biodiversity or air pollution that the population is exposed to, e.g., to identify vulnerable populations or to monitor the impact of mitigation and adaptation actions for climate resilience
	Gisler et al., 2021 (38)	Nose (nasal swabs)	47	16S rRNA V3-V5	Distinct microbiota profiles for different air pollution levels in the nasal microbiota of healthy infants	

### 4.1 Defining a “healthy” representative microbiome

To date, several hundred thousand microbial genomes have been reported in the human microbiomes, originating from over 5,000 different species (74), of which an estimated 50% is still undefined (75). In addition, a high degree of inter-individual variation has been observed (76). Therefore, the first step to enable population-based microbiome studies is to obtain an image of the general population’s microbiome by sampling the general public, without specific focus on certain (known) diseases, and thus representing a more or less “healthy” microbiome. This makes it possible to identify and study the diversity of species present in the microbiome of the country. Such projects are currently underway in several countries or regions (34, 77, 78) These projects are all based on convenience sampling with no random selection of participants from the “healthy” population (i.e., participation in these studies was at participant’s own initiative) and are therefore not representative of the general, national population. These samplings need to be conducted randomly at all levels of the population that is studied to cover not only different genders, ages and diets but also the diversity of socioeconomic status, ethnicities, childhood conditions or social behaviors (79). Indeed, most studies have tested a particularly high proportion of highly educated males (79), while it has previously been shown that the lack of representation of certain minority groups within the subjects tested can bias the interpretation of the results when applied to a more diverse group (80), as has been the case with the human genome (81). Including microbiome sampling in national health studies would allow to obtain information which is more representative of the general population, although some bias may remain due to non-response of some invited participants. Ensuring the representation of minorities among the participants could be attempted by oversampling of usually underrepresented groups, replacing non-respondents with people belonging to the same category, offering incentives (e.g., vouchers) or by adapting communication strategies (e.g., adapted study material, multiple languages, simplified languages, pre-testing of the communication material in a minority group) in order to ensure acceptability for specific minority groups that usually show a lower positive response to studies (82, 83). In addition, downstream analytical methods (e.g., weighting) can be used to aim for results as representative as possible.

### 4.2 Biological data from the microbiome in population-based health studies: added value

In previous studies (Table 1) alterations in the microbiomes have been associated with several parameters that could be measured during population-based health studies, such as diet, mental health or exposure to pollution. Population-based health studies cover demographic, clinical and/or exposure data. When combined with microbiome analysis, it would allow to gather biological evidence for a variety of factors in the population. Sampling of the microbiome could be included in ongoing population-based health studies such as food consumption surveys, human biomonitoring studies related to environmental chemical exposure (measurement of chemical concentrations in human samples), mental health assessments or projects related to climate change and environmental biodiversity alterations. Associations of this type of data with the microbiome have previously been reported for smaller population groups (Table 1). However, although it is generally assumed that microbiome shifts are observed in association with various lifestyle or health parameters, the causality link is often not established (10–12), and different studies may report contradicting biomarkers. The study of larger groups at different time points may address this issue (11, 84), as suggested by collaborations with national population-based health studies.

Through population-based health surveys, microbiome data could be associated with donor characteristics such as smoking status, region of residence and hence pollution data, diet or hygiene (Figure 1). Artificial intelligence, particularly machine learning and deep learning approaches, has been described as an interesting new tool for predicting health or exposure status (75, 85), but the population size needs to be sufficient to draw robust conclusions (86). Such associations could provide insights for public health policy and guidelines recommendations (Figure 1; Table 1). However, spurious associations (confounding effects) may be obtained if insufficient donor characteristics are collected. It is therefore important to collect sufficient donor information to limit this effect. Gender, age and body mass index have been shown to be important to include, as well as other specific factors depending on the type of microbiome being studied (87). Nevertheless, as unobserved data can also lead to these spurious associations, specific analysis methods that are available in the field of causal inference should be used to try to account for this.

National public health institutes can also sometimes have access to population cohorts for their studies. These are representative groups of people who are followed up year after year for environmental and health-related aspects. This allows longitudinal studies to be conducted on a random panel of the population. Longitudinal studies of cohorts have already been conducted to understand the evolution of the microbiome over time, particularly in the first years of life (3, 88, 89). Studying the microbiome of cohorts over time would also make it possible to uncover, at individual level (since the healthy microbiome varies greatly from person to person), but on a large scale, the dynamics related to the development and progression of diseases, the impact of pre-and post-exposure to pollution, the association with climate change as well as to observe at the biological and physiological level the impact of the implementation of new public health policies aimed at mitigating and adapting to certain adverse health and/or environmental impacts (e.g., air pollution, chemical exposure, loss of biodiversity).

Finally, other types of epidemiological data collection for microbiomes could be envisaged, beyond population surveys or cohorts. Indeed, human biomarkers can be studied at the population level in non-human samples such as sewage (90). For example, the gut microbiome detected in wastewater has previously been linked to obesity rates in the population (91). Taking advantage of the revised European Wastewater Treatment Directive (91/271/EEC), which requires the establishment of national wastewater-based surveillance by 2026, analyzing the sewage also through the lens of the microbiome it contains, would be an interesting, anonymized and non-invasive way to gather large-scale information at the population level and should be further investigated for its association with various exposures or health problems.

## 5 Discussion

The human microbiome has been studied for over a century and new high-throughput technologies have recently allowed to unravel an impact of environmental factors and medical conditions on these microbial communities but also hint to their influence on our health and well-being. However, such studies are not yet commonly conducted at the population level. Therefore, the integration of this biological data into health information systems has the potential to first define a representative microbiome of the general (“healthy”) population, and then, based on further research, to establish microbiome-based biomarkers that can be associated with exposures, health or population groups at risk. However, the integration of microbiome data into population-based health studies is complex and there are challenges to be overcome for the success of such large-scale studies, some of which have already been mentioned above such as standardization and the inclusion of minority groups. Another challenge is the high cost of advanced analytical methods. Although sequencing of the entire genetic material of a microbiome (shotgun metagenomics) would be ideal for higher accuracy and comparability of the taxonomic classification and even SNP-level information, metabarcoding is more cost-effective for a general idea of the species present in a sample. However, once biomarkers can be confirmed at the population level, some studies may only need to analyze a subset of species or genes using less advanced methods, further reducing costs. Proper data management will also be critical. Microbiome information will need to be collected anonymously in databases, along with the relevant additional donor characteristics. This should be harmonized within and between studies to avoid bias or errors in downstream analysis and interpretation. Subsequently, (bio) informatics tools will need to be developed, possibly using artificial intelligence-based methods to mine the data, to produce informed reports based on biological data, human biomonitoring, the exposome (including health behavior parameters) and population health to guide new policies. As discussed elsewhere (45), this will also require training of public health stakeholders in the interpretation of microbiome-based data.

Particularly at a time when various mitigation and adaptation actions measures are being implemented to reverse or cope with some of the detrimental anthropogenic environmental impacts of the past, microbiome data would eventually provide competent authorities with informed data on the whole population to monitor and evaluate the impact of these implemented actions. Therefore, including microbiome characterization in population-based studies is meaningful to further our knowledge of how microbiological data could be used in the future to assess health outcomes and environmental exposures.
